# Increasing trends in mortality and costs of infectious diseases in Korea: trends in mortality and costs of infectious diseases

**DOI:** 10.4178/epih.e2022010

**Published:** 2022-01-03

**Authors:** Dahye Baik, Byung-Woo Kim, Moran Ki

**Affiliations:** Department of Cancer Control and Population Health, Graduate School of Cancer Science and Policy, National Cancer Center, Goyang, Korea

**Keywords:** Infectious diseases, Mortality, Cost of illness, Trends, Republic of Korea

## Abstract

**OBJECTIVES:**

In an era when the average life expectancy and overall mortality rate have improved, Korea remains at risk for infectious disease outbreaks that place substantial burdens on the healthcare system. This study investigated trends in mortality and the economic burden of infectious diseases.

**METHODS:**

Healthcare data from the Health Insurance Review and Assessment Service (2009-2019) and the Korean Statistics Information Service (1997-2019) were used. We selected 10 infectious disease groups (intestinal infections, tuberculosis, vaccine- preventable diseases, sepsis, viral hepatitis, HIV-related diseases, central nervous system infections, rheumatic heart diseases, respiratory tract infections, and arthropod-borne viral diseases).

**RESULTS:**

The age-standardized mortality rate for infectious diseases increased from 27.2 per 100,000 population in 1997 to 37.1 per 100,000 population in 2019 and has had an upward trend since 2004. During this same period, significant increases were seen in respiratory tract infections and among elderly persons, especially those aged ≥85 years. The costs for infectious diseases increased from 4.126 billion US dollar (USD) in 2009 to 6.612 billion USD in 2019, with respiratory tract infections accounting for 3.699 billion USD (69%). The annual cost per patient for visits for medical care due to infectious diseases increased from 131 USD in 2009 to 204 USD in 2019.

**CONCLUSIONS:**

Mortality among elderly persons and those with respiratory tract infections increased during the study period. The economic burden of infectious diseases has consistently increased, especially for respiratory tract infections. It is therefore essential to establish effective management policies that considers specific infectious diseases and patient groups.

## INTRODUCTION

After industrialization, deaths caused by infectious diseases have decreased in many developed countries due to improvements in hygiene, the introduction of vaccines, and antimicrobial therapies [1]. However, the epidemiology of infectious diseases is complicated by socio-demographic factors, and infectious diseases still cause a considerable number of deaths globally [2]. Over the past few decades, the average life expectancy and overall mortality rate have improved in Korea, affected by rapid economic growth. The overall crude mortality rate decreased substantially from 6.4 deaths per 1,000 population in 1983 to 5.7 deaths per 1,000 population in 2019, as the average life expectancy rose from 67.5 years in 1983 to 83.2 years in 2019 [3,4]. However, Korea remains vulnerable to outbreaks of emerging infectious diseases that cause substantial burdens for the healthcare system, including severe acute respiratory syndrome in 2002-2003, H1N1 influenza in 2009, Middle East respiratory syndrome in 2015, and the ongoing outbreak of coronavirus disease 2019 (COVID-19) [5]. Moreover, some infectious diseases such as hepatitis B or hepatitis C virus and human papillomavirus affect the incidence of chronic diseases (e.g., cancer). With the rise in chronic diseases, most healthcare policies and financial resources have been focused on the management of non-communicable diseases in Korea [5]. Therefore, effective intervention strategies for managing infectious diseases are needed, as well as an exploration of changes in infectious disease epidemiology.

A cost-of-illness (COI) study is considered to be an essential measurement technique for evaluating the socioeconomic impacts of diseases. As a good baseline measure of the efficacy of health policies or intervention strategies, COI research can help healthcare decision makers determine and prioritize healthcare policies and interventions [6]. Research on the costs of infectious diseases has primarily focused on specific diseases such as hepatitis B and respiratory diseases [7,8]. Previous studies have also focused on disability-adjusted life years for communicable diseases [5] and the mortality trends of infectious diseases [1,9]. Research encompassing overall infectious diseases is scarce, and research concerning both mortality and the COI for infectious diseases has never been conducted in Korea. Therefore, this study aims to suggest priorities for evidence-based policy-making to control infectious diseases effectively by identifying trends in mortality and the economic burden of infectious diseases.

## MATERIALS AND METHODS

Our study used national death certificate data and the population census provided by the Korean Statistics Information Service (KOSIS) from 1997 to 2019, as well as healthcare statistics from the Health Insurance Review and Assessment Service (HIRA) between 2009 and 2019. In Korea, the National Health Insurance Service (NHIS) provides a mandatory insurance, with 97% of the population enrolled in its National Health Insurance program [7,10]. Moreover, its data include information from claims for reimbursed medical services, which contain details of the diseases and costs, records of inpatient and outpatient usage (diagnosis, length of stay, treatment costs), and prescription records (drug code, days prescribed, daily dosage), classified according to disease diagnosis [11].

Cases of infectious diseases were selected based on the primary diagnosis and confirmed when inpatient and outpatient claims occurred at least once over the course of one year. With reference to the study by Choe et al. [1], the cases in our study were classified into 10 disease groups (Table 1). The International Statistical Classification of Disease and Related Health Problems 10th revision (ICD-10) code was used to identify each infectious disease.

The mortality rate was defined as the annual number of deaths caused by infectious diseases (individually and overall), based on the cause of death data from KOSIS, divided by the total population, and using KOSIS data on sex and age. The prevalence rate was defined as the number of patients who were treated for an infectious disease each year. The annual total number of infectious disease cases from the HIRA data, divided by the total registered population, was also used. The age-standardized mortality rate (ASMR) and age-standardized prevalence rate (ASPR) were adjusted using registered population data from 2010. Both rates are shown as the rate per 100,000 people. To identify the trend of the mortality rate, average percent change (APC) analyses were performed using the Joinpoint Regression Program version 4.8.0.1 (Nation al Cancer Institute, Bethesda, MD, USA).

The costs of infectious diseases were estimated using a prevalence-based approach, which estimates the economic burden of a condition over a specific period, usually a year [6]. The total annual costs of treatment were measured among both pre-existing and newly diagnosed patients, regardless of the number of visits to seek medical care by the same patient. For this study, direct healthcare costs were considered the total healthcare cost including detailed healthcare utilization, prescribed drugs, and medical costs. The costs were calculated by the sum of medical costs paid by inpatients and outpatients at medical institutions and pharmacies. In this study, 1 US dollar (USD) equaled 1,136.7 Korean won, as determined by the average exchange rate between 2009 and 2019 [12]. To eliminate the effects of inflation, annual costs were adjusted based on 2015 costs using the Korean Consumer Price Index data by the expenditure category of health [13].

### Ethics statement

This study was approved by the Institutional Review Board of National Cancer Center (IRB No. NCC2020-0034).

## RESULTS

**Figure 1.**
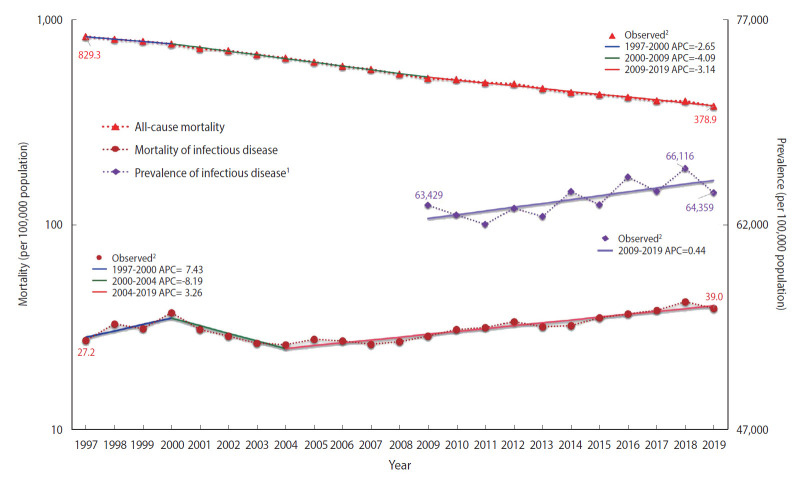
Age-standardized mortality (1997-2019) and prevalence (2009-2019) rates of infectious diseases. ^1^Prevalence of infectious disease was considered as the annual total number of patients who were treated for infectious disease; It was adjusted using registered population data in 2010. ^2^indicates that average percent change (APC) is significantly different from zero at the α=0.05 level.

The overall ASMR in Korea had a negative slope, from 829.3 per 100,000 population in 1997 to 378.9 per 100,000 population in 2019 (APC, -3.5%, p<0.05). Nevertheless, the number of deaths caused by infectious diseases increased from 8,292 in 1997 to 33,600 in 2019. The ASMR of all infectious diseases increased from 27.2 per 100,000 population in 1997 to 37.1 per 100,000 population in 2000 (APC, 7.43%), but then declined annually by 8.19% (p<0.05) to 26.0 per 100,000 population in 2004 (Figure 1). After 2004, it gradually increased to 39.0 per 100,000 population in 2019 (APC, 3.26%, p<0.05). Although the ASPR of all infectious diseases fluctuated annually, it exhibited an upward trend from 63,429 per 100,000 population in 2009 to 64,359 per 100,000 population in 2019. The average annual percent change (AAPC) was 1.60% in ASMR (p<0.05) and 0.44% in ASPR (p<0.05).

**Figure 2.**
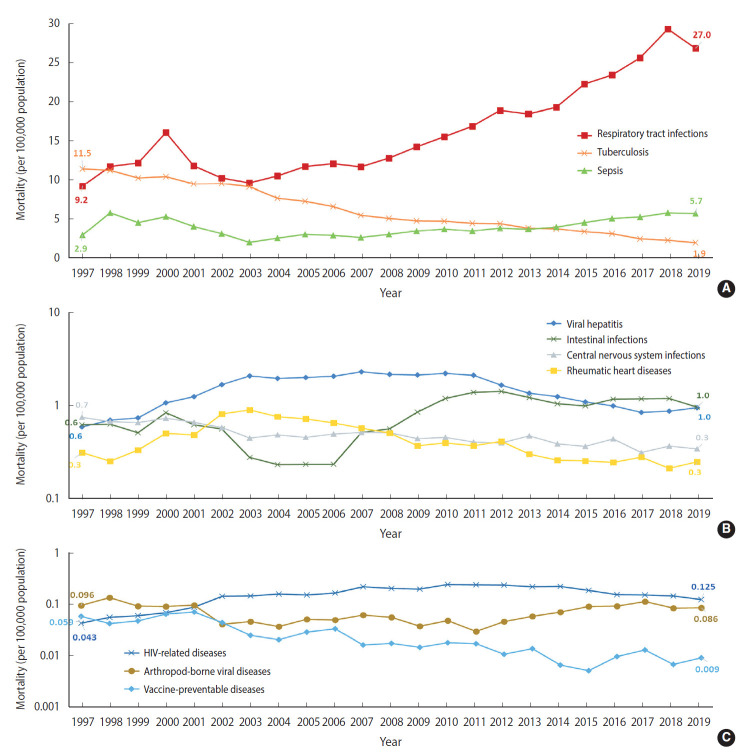
Age-standardized mortality rate of infectious diseases by 10 selected groups in Korea, 1997-2019. Age-standardized mortality rate associated with respiratory tract infections, tuberculosis, and sepsis (A), viral hepatitis, intestinal infections, central nervous system infections, and rhuematic heart diseases (B), and HIV-related diseases, arthropod-borne viral disease, and vaccine-preventable diseases (C).

In 1997, tuberculosis was the leading cause of infectious disease deaths; the ASMR was 11.5 per 100,000 population (Figure 2). However, the ASMR from tuberculosis has gradually declined since 1997 and was 1.9 per 100,000 population in 2019 (AAPC, -7.7%, p<0.05) (Supplementary Material 1). Respiratory tract infections have been the leading cause of infectious disease deaths since 1998. Moreover, there was a dramatic rise in ASMR from respiratory tract infections, rising from 9.2 per 100,000 population in 1997 to 27.0 per 100,000 population in 2019 (AAPC, 5.2%, p<0.05) (Figure 2 and Supplementary Material 1). The ASMR from sepsis has shown an increasing trend since 2003, and sepsis has been the second leading cause of infectious disease deaths since 2014. In 1997 the ASMR from central nervous system infections was 0.7 per 100,000 population, followed by intestinal infections and viral hepatitis (each 0.6 per 100,000 population). However, viral hepatitis has become the fourth leading cause of infectious disease deaths; the ASMR from viral hepatitis was 2.0 per 100,000 population in 2015, followed by intestinal infections at 1.0 per 100,000 population and central nervous system infections at 0.4 per 100,000 population. The ASMR ranks of viral hepatitis and intestinal infections were finally reversed as of 2016.

The ASPR of respiratory tract infections was highest among the 10 disease groups during the study period (2009-2019). Annually, an average 54,449 per 100,000 population were diagnosed with respiratory tract infections (Supplementary Material 2). Intestinal infection was the second most frequent diagnosis among the 10 disease groups, followed by viral hepatitis. As the ASPR from tuberculosis dramatically decreased, it declined from 235.9 per 100,000 population in 2009 to 86.8 per 100,000 population in 2019.

**Figure 3.**
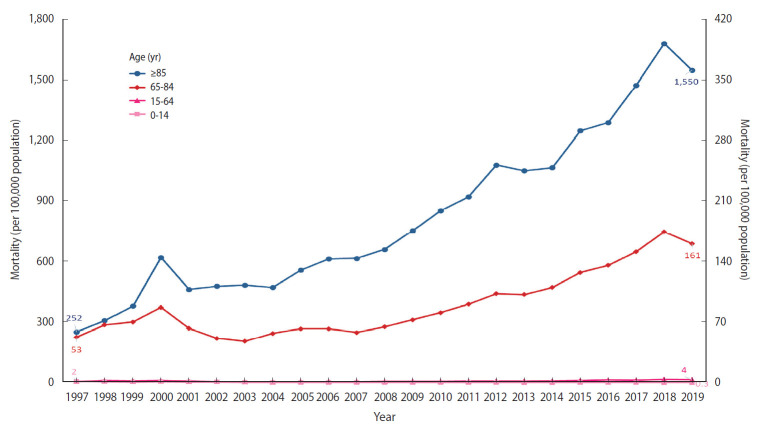
Age-specific mortality rate of respiratory tract infections in Korea, 1997-2019.

Regarding respiratory tract infections, there are significant differences in the ASMR among the age groups (Figure 3). Whereas the mortality rate in the young age group (0-14 years) decreased during the study period (1997-2019), the mortality rate in the old age group (+65 years) consistently increased. Notably, the rate for those aged ≥ 85 years increased dramatically from 252 deaths per 100,000 population in 1997 to 1,550 deaths per 100,000 population in 2019.

**Figure 4.**
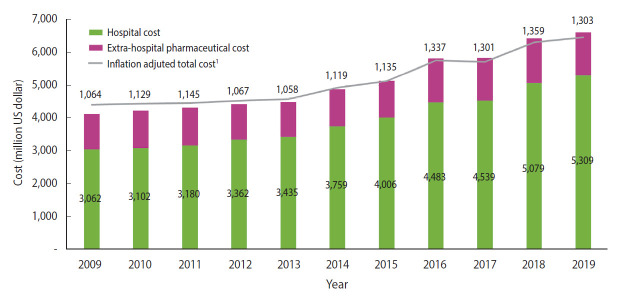
Trends in healthcare costs for infectious diseases in Korea, 2009-2019. ^1^Adjusted using Consumer Price Index in 2015 [13].

The total healthcare costs of infectious diseases showed an upward trend during the study period (2009-2019) in Korea (Figure 4). The cost was 4,126 million USD in 2009 including pharmaceutical costs (1,064 million USD). In 2019, the cost was 1.6 times higher, at 6,612 million USD including pharmaceutical costs (1,303 million USD). The inflation-adjusted total costs also increased during the period.

**Figure 5.**
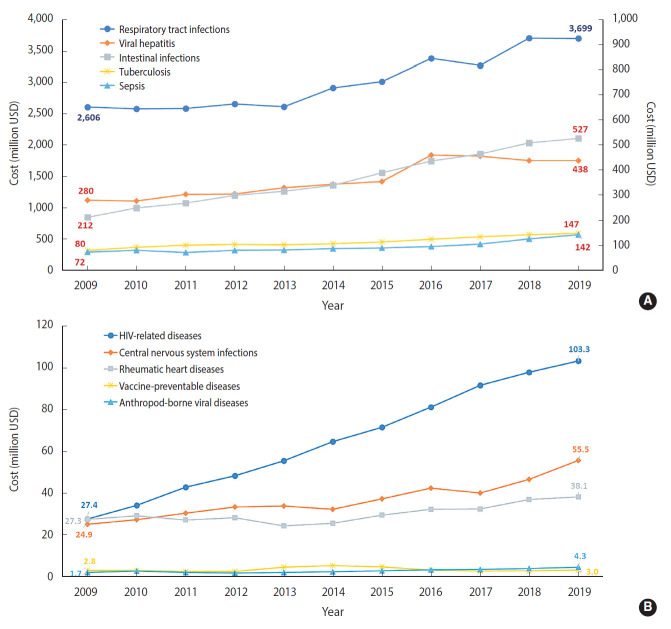
Cost of infectious diseases by 10 selected groups in Korea, 2009-2019. Cost associated with respiratory tract infections, viral hepatitis, intestinal infections, tuberculosis, and sepsis (A), HIV-related diseases, central nervous system infections, rheumatic heart diseases, vaccine-preventable diseases, and arthropod-borne viral diseases (B). USD, US dollar.

Respiratory tract infections accounted for the highest total costs among the 10 disease groups during the study period (2009-2019), with 2,606 million USD in 2009 and 3,699 million USD in 2019, reflecting a 1.4-fold increase (Figure 5). In 2009, viral hepatitis had the second-highest medical costs of the 10 disease groups, followed by intestinal infections. Conversely, in 2019 intestinal infections had the second-highest medical costs, followed by viral hepatitis. Costs from the other disease groups have shown increasing trends. Among those groups, the costs from HIV-related diseases had the highest rate of increase during the period, going from 27.4 million USD in 2009 to 103.3 million USD (3.8 times greater) in 2019.

In 2009, 31,570,316 patients were treated for infectious diseases, increasing to 32,374,393 patients in 2019 (Table 2). The healthcare cost per patient was 131 USD in 2009 and was 1.6 times higher in 2019 at 204 USD. Taking into account the effect of inflation, both total healthcare costs and average costs per patient have increased during the same period. With respect to both inpatient and outpatient infectious disease cases, both the number of patients and the average cost per patient have increased from 2009 to 2019. The average length of hospitalization was 9 days, and the average number of visits for medical care was 5.3 during the study period (2009-2019).

## DISCUSSION

This study analyzed mortality and healthcare cost trends for infectious diseases in Korea, using nationally representative KOSIS data and NHIS data from HIRA. Although the ASMR from all-cause deaths has declined, the ASMR and ASPR of infectious diseases have trended upward (AAPC,1.6%, p<0.05; AAPC, 0.44%, p<0.05, respectively). The healthcare costs of infectious diseases also showed a positive slope.

The ASMR from respiratory tract infections showed a 3-fold increase during the study period. In 1997, respiratory tract infectious accounted for 34% of deaths caused by infectious diseases, while in 2019 the proportion was 2 times higher at 69%. Deaths were attributed to pneumonia and were highest in older age groups, especially those 85 years and older. Although the ASPR from sepsis decreased slowly, the ASMR from sepsis has increased since 2003. This suggests that respiratory tract infections (particularly pneumonia) and sepsis have become the leading causes of death due to infectious diseases with the rapid aging of the Korean population.

Although the ASMR and ASPR from tuberculosis have consistently declined, Korea was listed as the country with the highest incidence and the second-highest mortality rate for tuberculosis among the 36 Organization for Economic Cooperation and Development countries in 2019 [14]. The healthcare cost for treating tuberculosis has also increased. Although the ASMR from viral hepatitis was not high (average 1.5 per 100,000 population,1997-2019), the ASPR from viral hepatitis was the third most diagnosed infectious disease among the 10 disease groups (average 880.7 per 100,000 population, 2009-2019). This has resulted in increased healthcare costs, mainly due to the use of antiviral drugs [7]. Moreover, the related chronic diseases caused by viral hepatitis frequently occur. It suggests that tuberculosis and viral hepatitis, especially hepatitis B and hepatitis C infections, still remained a heavy healthcare burden for Korea.

Annually, 63.4% of Koreans sought medical care for the treatment of infectious diseases (2009-2019). Most of them were outpatient visits, while an average of 3.6% of these patients were inpatients. During the study period, 3.2% of the total patients with infectious diseases received both outpatient and inpatient care during the study period. The average days of hospitalization decreased and the number of outpatient visits gradually declined over the study period. However, the total costs and the average cost per patient caused by infectious diseases showed upward trends. This demonstrates that the financial burden of healthcare, specifically the costs for treating and managing patients with infectious diseases, has significantly increased in Korea.

A strength of this study was its use of NHIS claims data, which are representative of the nationwide population. Therefore, its results represent the data from all patients with infectious diseases in Korea. Moreover, this study identified the costs of infectious diseases for the first time in Korea. These findings should be used as important reference data for estimating the burden of infectious diseases in Korea before the COVID-19 outbreak. In 2019, the total medical expenditures from the HIRA report were 75.684 billion USD [15]. The healthcare cost for infectious diseases accounted for 8.7% of this amount, lower than that of 2009 (11.9%). However, it could not be concluded that the burden of infectious diseases has decreased because of the annual changes in total medical expenditures. These changes resulted from annual changes in reimbursement policies and population aging. It is difficult to calculate the exact proportion of the burden of infectious diseases, but the trends will be changed by the COVID-19 outbreak. This study will be an important reference for comparing the changes in epidemiology and burden of infectious diseases before and after the outbreak of COVID-19. Further research should consider the mortality and costs of infectious diseases since 2020.

However, there are some limitations to this study. First, the costs were likely underestimated because this study only considered cases where an infectious disease was the primary diagnosis. Secondly, the practical classification of infectious diseases might not reflect changes in disease epidemiology and the specific public health measures for control and prevention, including changes in vaccination coverage [1]. For example, influenza, hepatitis A, and hepatitis B are now recognized as diseases that can be prevented by vaccination, but influenza is still classified as respiratory tract infection, and hepatitis A and hepatitis B are still classified as viral hepatitis. Finally, because the healthcare statistics of HIRA were used to estimate the cost of infectious diseases, this study could not identify detailed cost categories. Future research that considers age groups; costs by socioeconomic status, region, and service category; medication or prescription costs; doctor’s fees; and admission charges is needed to set priorities for the effective control and prevention of infectious disease.

In conclusion, the mortality trends (1997-2019) and costs (2009-2019) of infectious diseases in Korea have increased. In particular, respiratory tract infections require attention from disease control experts and agencies because of the high mortality rates in elderly persons with respiratory tract infections and the high medical costs of treating them as compared to the costs of treating the other 10 disease groups. This study highlights the need for effective policies and efficient management for each disease group to reduce the burden of infectious diseases. Moreover, this study is a particularly important reference for identifying long-term trends, including mortality, infectious disease outbreaks, and the financial burden of infectious diseases in Korea before the COVID-19 outbreak.

## Figures and Tables

**Table 1.** Selection of infectious disease groups using the International Statistical Classification of Disease and Related Health Problems 10th revision (ICD-10) codes

Groups		ICD-10 codes
All infectious diseases		A00-B99, G00-G09, I00-I09, J00-J06, J09-J18, J20-J22, J85-J86, L00-L08, M86, N10-N12, N70-N77, P35-P39
	Intestinal infections	A00-A09
	Tuberculosis	A15-A19
	Vaccine-preventable diseases (includes diphtheria, tetanus, pertussis, measles, mumps)	A33-A37, A39, B05, B06, B26
	Sepsis	A40-A41
	Arthropod-borne viral diseases (including mosquito-borne and tick-borne viral encephalitis, viral [hemorrhagic] fevers, malaria)	A83, A84, A92-A99, B50-B54
	Viral hepatitis	B15-B19
	HIV-related diseases	B20-B24
	Central nervous system infections	G00-G09
	Rheumatic heart diseases	I00-I09
	Respiratory tract infections (including influenza, pneumonia, empyema)	J00-J06, J09-J18, J20-J22, J85-J86

**Table 2.** The number of patients and the costs of infectious diseases in Korea, 2009-2019

Year	No. of patients (person)	Inpatient					Outpatient					Total cost (million USD)	Cost per patient (USD)^1^	Inflation adjusted total cost (million USD)^2^
		No. of patients (person)	Days of hospitalization (day)	Average days of hospitalization (day/person)	Cost (million USD)	Average cost per patient (USD)	No. of patients (person)	Days of visit (day)	Average days of visit (day/person)	Cost (million USD)	Average cost per patient (USD)			
2009	31,570,316	870,586	8,338,531	9.6	922	1,059	31,481,267	168,665,913	5.4	3,204	102	4,126	131	4,412
2010	31,286,710	943,115	9,068,217	9.6	1,026	1,088	31,190,895	170,266,963	5.5	3,205	103	4,231	135	4,446
2011	31,056,053	977,903	9,340,405	9.6	1,092	1,117	30,718,861	165,983,722	5.4	3,232	105	4,325	139	4,466
2012	31,733,206	1,038,167	9,727,210	9.4	1,154	1,111	31,629,989	171,376,085	5.4	3,275	104	4,429	140	4,532
2013	31,508,376	1,027,771	9,666,999	9.4	1,178	1,147	31,399,997	167,566,340	5.3	3,314	106	4,492	143	4,581
2014	32,485,239	1,123,732	10,315,378	9.2	1,302	1,159	32,371,607	173,371,141	5.4	3,576	110	4,878	150	4,939
2015	32,069,591	1,211,936	11,047,023	9.1	1,510	1,246	31,944,506	166,382,056	5.2	3,631	114	5,141	160	5,141
2016	33,082,602	1,506,536	11,964,776	7.9	1,742	1,156	32,927,734	176,992,342	5.4	4,078	124	5,820	176	5,763
2017	32,571,529	1,349,999	11,124,243	8.2	1,785	1,322	32,411,697	167,472,742	5.2	4,054	125	5,840	179	5,732
2018	33,416,385	1,437,389	11,846,714	8.2	2,090	1,454	33,249,884	171,840,247	5.2	4,348	131	6,438	193	6,322
2019	32,374,393	1,386,980	11,677,868	8.4	2,278	1,643	32,210,573	164,638,317	5.1	4,334	135	6,612	204	6,464

USD, US dollar.

1 Cost per patient=Total cost of the 2009-2019 period/number of patients who received treatment for infectious disease in the 2009-2019 period.

2 Inflation adjusted costs were adjusted using the Consumer Price Index in 2015 [13].

## SUPPLEMENTARY MATERIALS

Supplementary Material 1. Trends in age-standardized mortality rate of infectious disease by 10 selected groups using Joinpoint analyses in Republic of Korea, 1997-2019

Supplementary Material 2 Age-standardized prevalence rate of infectious disease by 10 selected groups in Republic of Korea, 2009-2019

## References

[1] Choe YJ, Choe SA, Cho SI (2018). Trends in infectious disease mortality, South Korea, 1983-2015. Emerg Infect Dis.

[2] GBD 2015 Mortality and Causes of Death Collaborators (2016). Global, regional, and national life expectancy, all-cause mortality, and cause-specific mortality for 249 causes of death, 1980-2015: a systematic analysis for the Global Burden of Disease Study 2015. Lancet.

[3] World Bank Death rate, crude (per 1,000 people) - Korea, Rep. https://data.worldbank.org/indicator/SP.DYN.CDRT.IN?locations=KR.

[4] World Bank Life expectancy at birth, total (years). https://data.worldbank.org/indicator/SP.DYN.LE00.IN?location=KR.

[5] Lee YR, Moon K, Kim YA, Park SY, Oh CM, Lee KS (2016). Disability-adjusted life years for communicable disease in the Korean burden of disease study 2012. J Korean Med Sci.

[6] Jo C (2014). Cost-of-illness studies: concepts, scopes, and methods. Clin Mol Hepatol.

[7] Baik D, Kim BW, Oh JK, Kim KA, Ki M (2020). Costs of viral hepatitis B in the Republic of Korea, 2002-2015. J Viral Hepat.

[8] Yoo KH, Ahn HR, Park JK, Kim JW, Nam GH, Hong SK (2016). Burden of respiratory disease in Korea: an observational study on allergic rhinitis, asthma, COPD, and rhinosinusitis. Allergy Asthma Immunol Res.

[9] Kim HS, Eun SJ (2021). Age-period-cohort analysis of trends in infectious disease mortality in South Korea from 1983 to 2017. Int J Environ Res Public Health.

[10] Kim HK, Song SO, Noh J, Jeong IK, Lee BW (2020). Data configuration and publication trends for the Korean National Health Insurance and Health Insurance Review & Assessment database. Diabetes Metab J.

[11] Seong SC, Kim YY, Khang YH, Park JH, Kang HJ, Lee H (2017). Data resource profile: the National Health Information Database of the National Health Insurance Service in South Korea. Int J Epidemiol.

[12] Korean Statistical Information Service (2021). Foreign exchange rate. https://kosis.kr/statHtml/statHtml.do?orgId=101&tblId=DT_2KAA811&conn_path=I3.

[13] Korean Statistical Information Service Consumer price index by item. https://kosis.kr/statHtml/statHtml.do?orgId=101&tblId=DT_1J20001&conn_path=I2.

[14] World Health Organization Global tuberculosis report 2019. https://www.who.int/publications/i/item/9789241565714.

[15] National Health Insurance Service (2020). National health insurance statistical yearbook 2020.

