# Supplementary material for: Increasing trends in mortality and costs of infectious diseases in Korea: trends in mortality and costs of infectious diseases
Source: Epidemiol Health. 2022 Jan 3;44:e2022010. doi: 10.4178/epih.e2022010 (PMC9117094; doi:10.4178/epih.e2022010)
Supplement: Supplementary Material 1. — Trends in age-standardized mortality rate of infectious disease by 10 selected groups using Joinpoint analyses in Republic of Korea, 1997-2019 [file epih-44-e2022010-suppl1.ppt]

## Slide 1
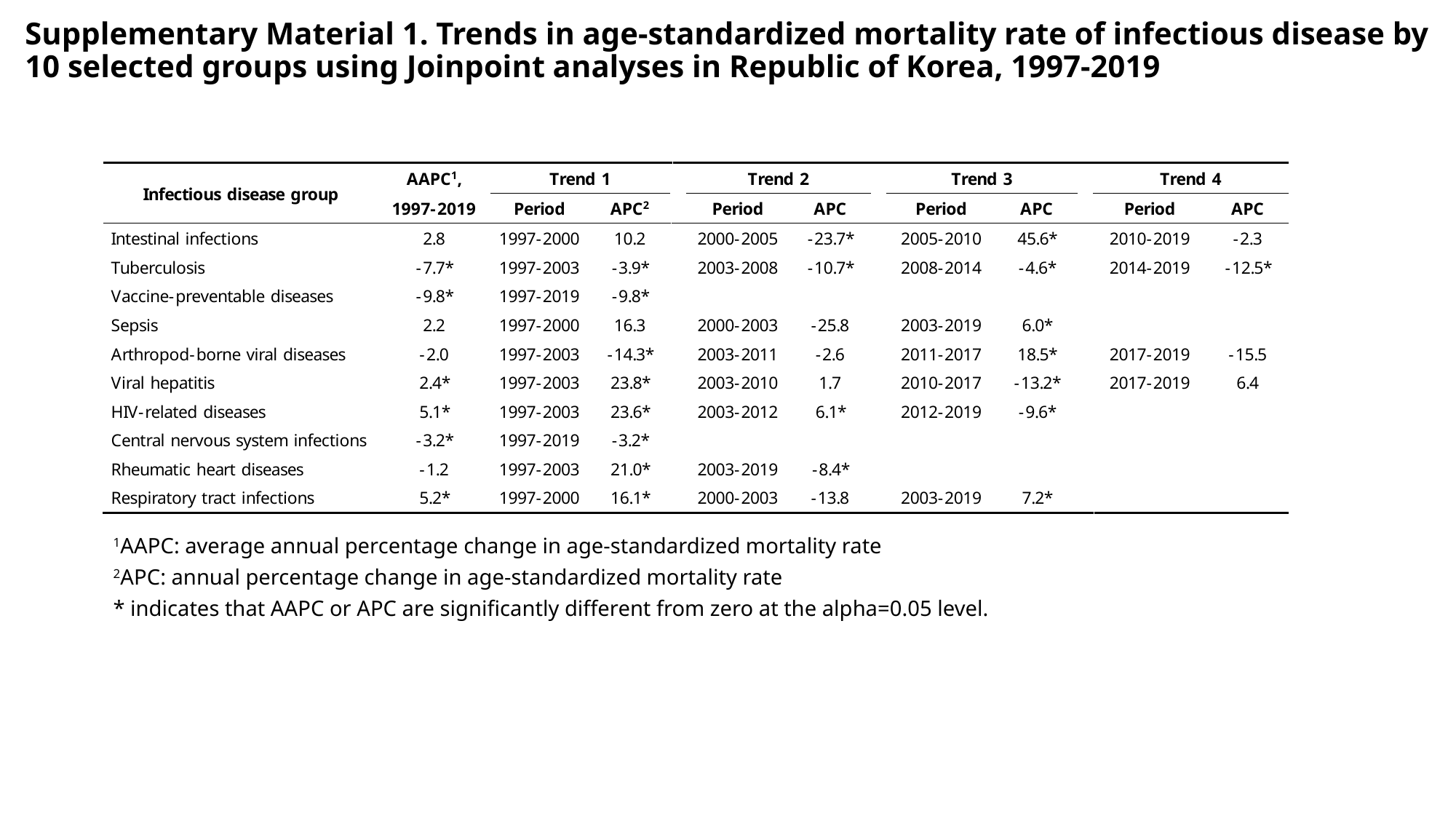

# Supplementary Material 1. Trends in age-standardized mortality rate of infectious disease by 10 selected groups using Joinpoint analyses in Republic of Korea, 1997-2019
1AAPC: average annual percentage change in age-standardized mortality rate
2APC: annual percentage change in age-standardized mortality rate
* indicates that AAPC or APC are significantly different from zero at the alpha=0.05 level.
