# Supplementary material for: Increasing trends in mortality and costs of infectious diseases in Korea: trends in mortality and costs of infectious diseases
Source: Epidemiol Health. 2022 Jan 3;44:e2022010. doi: 10.4178/epih.e2022010 (PMC9117094; doi:10.4178/epih.e2022010)
Supplement: Supplementary Material 2 — Age-standardized prevalence rate of infectious disease by 10 selected groups in Republic of Korea, 2009-2019 [file epih-44-e2022010-suppl2.ppt]

## Slide 1
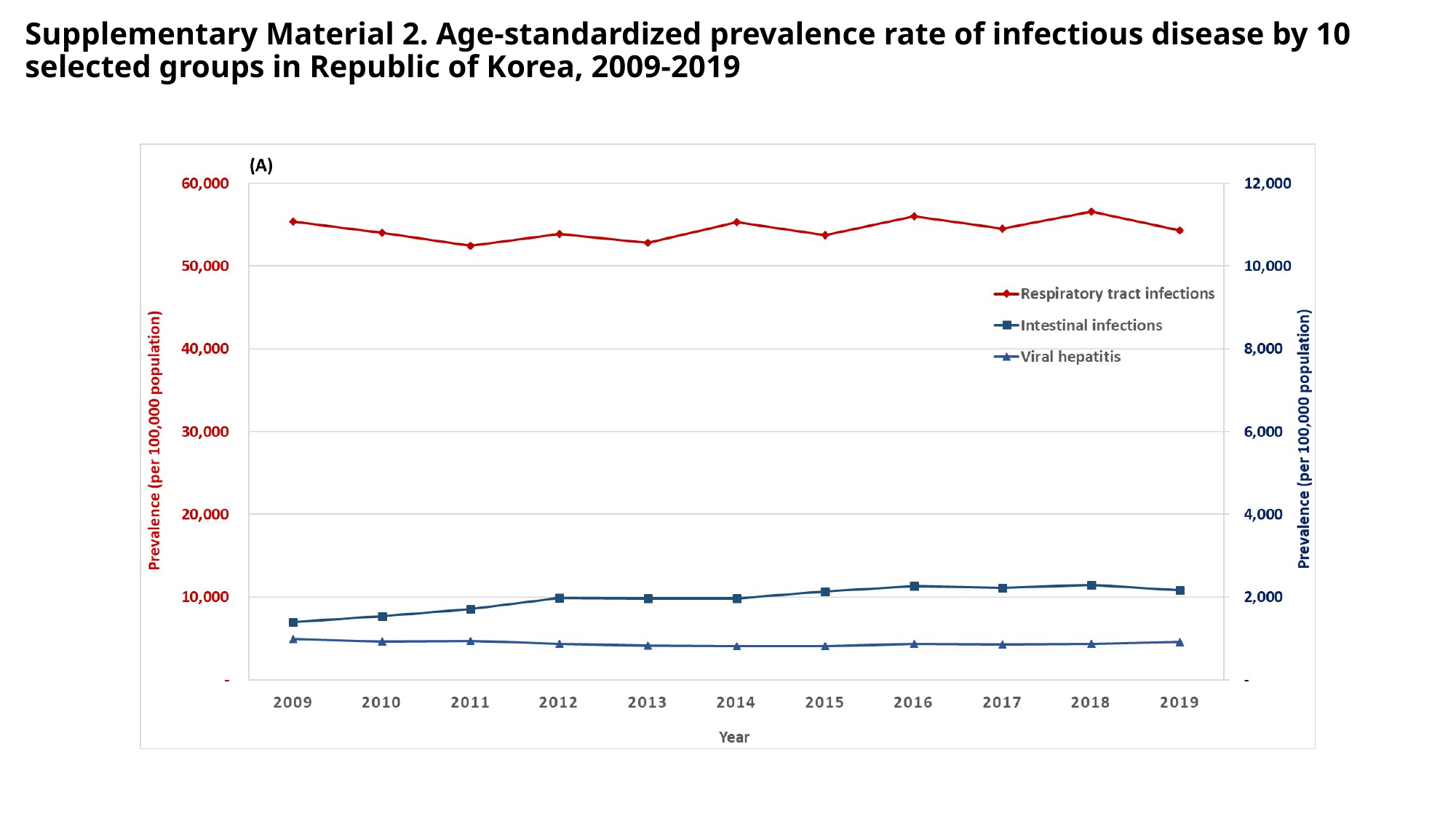

# Supplementary Material 2. Age-standardized prevalence rate of infectious disease by 10 selected groups in Republic of Korea, 2009-2019

## Slide 2
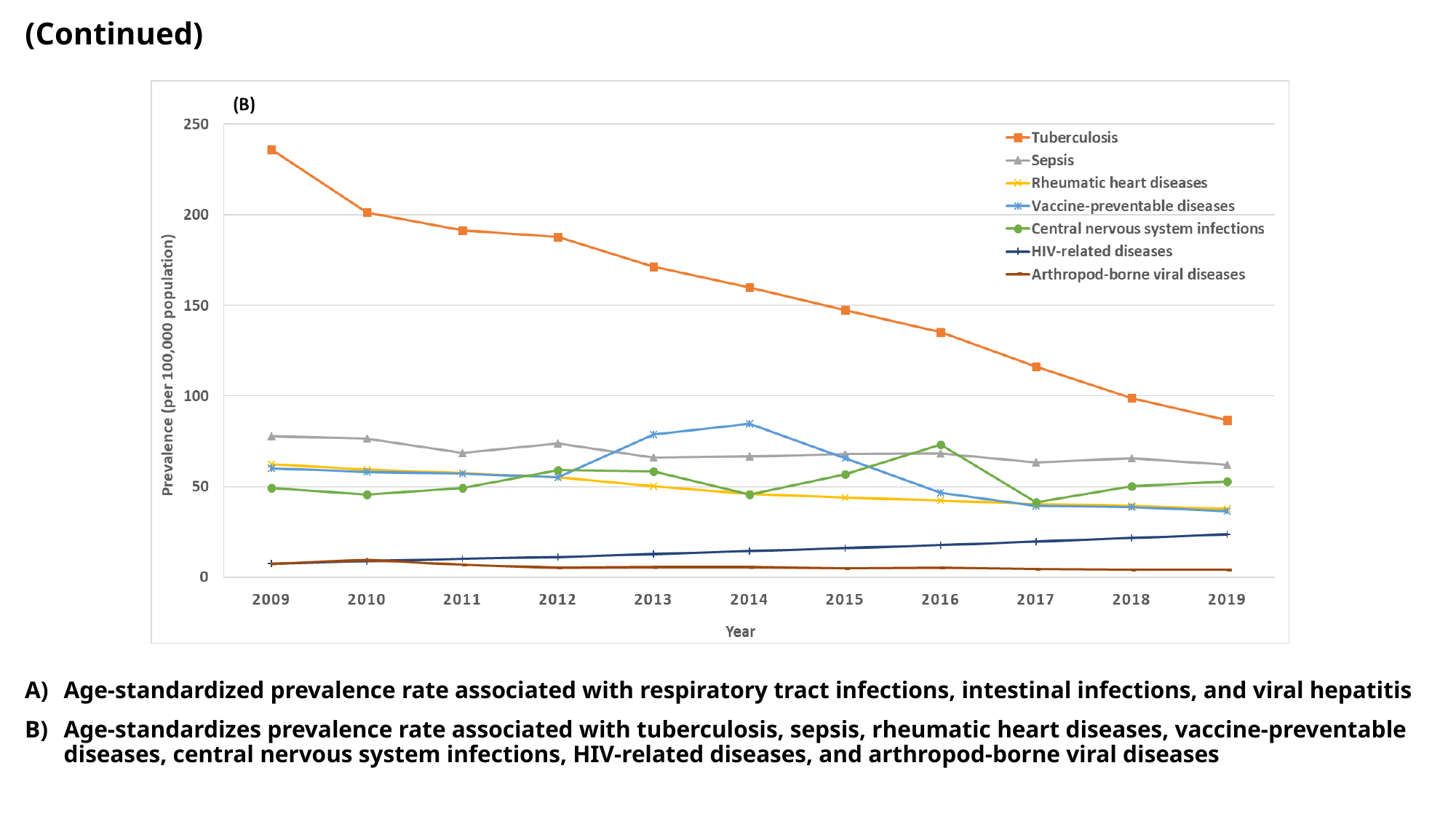

# (Continued)
Age-standardized prevalence rate associated with respiratory tract infections, intestinal infections, and viral hepatitis
Age-standardizes prevalence rate associated with tuberculosis, sepsis, rheumatic heart diseases, vaccine-preventable diseases, central nervous system infections, HIV-related diseases, and arthropod-borne viral diseases
